# Disease Progression of WHIM Syndrome in an International Cohort of 66 Pediatric and Adult Patients

**DOI:** 10.1007/s10875-022-01312-7

**Published:** 2022-08-10

**Authors:** Christoph B. Geier, Maryssa Ellison, Rachel Cruz, Sumit Pawar, Alexander Leiss-Piller, Katarina Zmajkovicova, Shannon M McNulty, Melis Yilmaz, Martin Oman Evans, Sumai Gordon, Boglarka Ujhazi, Ivana Wiest, Hassan Abolhassani, Asghar Aghamohammadi, Sara Barmettler, Saleh Bhar, Anastasia Bondarenko, Audrey Anna Bolyard, David Buchbinder, Michaela Cada, Mirta Cavieres, James A. Connelly, David C. Dale, Ekaterina Deordieva, Morna J. Dorsey, Simon B. Drysdale, Stephan Ehl, Reem Elfeky, Francesca Fioredda, Frank Firkin, Elizabeth Förster-Waldl, Bob Geng, Vera Goda, Luis Gonzalez-Granado, Eyal Grunebaum, Elzbieta Grzesk, Sarah E. Henrickson, Anna Hilfanova, Mitsuteru Hiwatari, Chihaya Imai, Winnie Ip, Soma Jyonouchi, Hirokazu Kanegane, Yuta Kawahara, Amer M. Khojah, Vy Hong-Diep Kim, Marina Kojić, Sylwia Kołtan, Gergely Krivan, Daman Langguth, Yu-Lung Lau, Daniel Leung, Maurizio Miano, Irina Mersyanova, Talal Mousallem, Mica Muskat, Flavio A. Naoum, Suzie A. Noronha, Monia Ouederni, Shuichi Ozono, G. Wendell Richmond, Inga Sakovich, Ulrich Salzer, Catharina Schuetz, Filiz Odabasi Seeborg, Svetlana O. Sharapova, Katja Sockel, Alla Volokha, Malte von Bonin, Klaus Warnatz, Oliver Wegehaupt, Geoffrey A. Weinberg, Ke-Juin Wong, Austen Worth, Huang Yu, Yulia Zharankova, Xiaodong Zhao, Lisa Devlin, Adriana Badarau, Krisztian Csomos, Marton Keszei, Joao Pereira, Arthur G Taveras, Sarah L. Beaussant-Cohen, Mei-Sing Ong, Anna Shcherbina, Jolan E. Walter

**Affiliations:** 1 Institute for Immunodeficiency, Center for Chronic Immunodeficiency, Medical Center University of Freiburg Faculty of Medicine, University of Freiburg, Freiburg, Germany; 2 Department of Rheumatology and Clinical Immunology, Center for Chronic Immunodeficiency, Medical Center University of Freiburg, Faculty of Medicine, University of Freiburg, Freiburg, Germany; 3 Division of Pediatric Allergy and Immunology, Department of Pediatrics, University of South Florida, St. Petersburg, FL USA; 4 Morsani College of Medicine, University of South Florida, Tampa, FL USA; 5 X4 Pharmaceuticals (Austria) GmbH, Vienna, Austria; 6 Immunology Outpatient Clinic, Vienna, Austria; 7 Department of Genetics, University of North Carolina at Chapel Hill, Chapel Hill, NC USA; 8 Blanchfield Army Community Hospital, Fort Campbell, KY USA; 9 Research Center for Immunodeficiencies, Pediatrics Center of Excellence, Children’s Medical Center, Tehran University of Medical Sciences, Tehran, Iran; 10 Division of Clinical Immunology, Department of Laboratory Medicine, Karolinska Institute at Karolinska University Hospital Huddinge, Stockholm, Sweden; 11 Allergy and Clinical Immunology Unit, Division of Rheumatology, Allergy & Immunology, Massachusetts General Hospital, Boston, MA USA; 12 Department of Pediatrics, Section of Hematology/Oncology and Critical Care Medicine, Bone Marrow Transplantation, Baylor College of Medicine, Texas Children’s Hospital, Houston, TX USA; 13 Shupyk National Medical Academy of Postgraduate Education, Kyiv, Ukraine; 14 Severe Chronic Neutropenia International Registry, University of Washington, Seattle, WA USA; 15 Division of Hematology, CHOC Children’s Hospital, Orange, CA USA; 16 Division of Hematology and Oncology, Hospital for Sick Children, Toronto, Ontario Canada; 17 Department of Pediatrics, University of Toronto, Toronto, Ontario Canada; 18 Hematology Unit, Dr Luis Calvo Mackenna Children’s Hospital, Santiago, Chile; 19 Vanderbilt University Medical Center, Nashville, TN USA; 20 Department of Medicine, University of Washington, Seattle, WA USA; 21 Immunology, the Dmitry Rogachev National Medical Center of Pediatric Hematology, Oncology and Immunology, Moscow, Russia; 22 Division of Allergy, Immunology and Blood and Marrow Transplantation, Department of Pediatrics, UCSF Benioff Children’s Hospital, San Francisco, CA USA; 23 Paediatric Infectious Diseases Research Group, St George’s University Hospitals NHS Foundation Trust, London, UK; 24 Department of Clinical Immunology, Royal Free Hospital, London, UK; 25 Haematology Unit, IRCCS Istituto Giannina Gaslini, Genoa, Italy; 26 Department of Medicine, St Vincent’s Hospital, University of Melbourne, Vic, Fitzroy, Australia; 27 Department of Clinical Haematology, St Vincent’s Hospital, Vic, Fitzroy, Australia; 28 Department of Pediatrics and Adolescent Medicine, Medical University of Vienna, Vienna, Austria; 29 Division of Neonatology, Pediatric Intensive Care & Neuropediatrics, Department of Pediatrics and Adolescent Medicine, Medical University of Vienna, Vienna, Austria; 30 Center for Congenital Immunodeficiencies, Medical University of Vienna & Jeffrey Modell Diagnostic and Research Center, Vienna, Austria; 31 Divisions of Adult and Pediatric Allergy and Immunology, University of California, San Diego, CA USA; 32 Department for Pediatric Hematology and Hemopoietic Stem Cell Transplantation, Central Hospital of Southern Pest - National Institute of Hematology and Infectious Diseases, Budapest, Hungary; 33 Immunodeficiencies Unit, Department of Pediatrics, University Hospital 12 de Octubre, Research Institute Hospital 12 Octubre, Madrid, Spain; 34 Division of Immunology and Allergy, Hospital for Sick Children, Toronto, Ontario Canada; 35 Department of Pediatrics, Hematology and Oncology Collegium Medicum, Bydgoszcz Nicolaus Copernicus University, Torun, Poland; 36 Division of Allergy and Immunology, Children’s Hospital of Philadelphia, Philadelphia, PA USA; 37 Department of Pediatrics, Perelman School of Medicine, University of Pennsylvania, Philadelphia, PA USA; 38 Institute for Immunology, Perelman School of Medicine, University of Pennsylvania, Philadelphia, PA USA; 39 Department of Pediatrics, Graduate School of Medicine, The University of Tokyo, Tokyo, Japan; 40 Department of Pediatrics, Niigata University Graduate School of Medical and Dental Sciences, Niigata, Japan; 41 Department of Pediatrics, Niigata University Medical and Dental Hospital, Niigata, Japan; 42 Great Ormond Street Hospital for Children, London, UK; 43 Division of Allergy and Immunology, Department of Pediatrics, Children’s Hospital of Philadelphia, University of Pennsylvania Perelman School of Medicine, Philadelphia, PA USA; 44 Department of Child Health and Development, Graduate School of Medical and Dental Sciences, Tokyo Medical and Dental University (TMDU), Tokyo, Japan; 45 Department of Pediatrics, Jichi Medical University School of Medicine, Tochigi, Japan; 46 Northwestern University Feinberg School of Medicine, Chicago, IL USA; 47 Department of Immunology, Sullivan and Nicolaides Pathology, Brisbane, Australia; 48 Department of Paediatrics and Adolescent Medicine, University of Hong Kong, Hong Kong, China; 49 Department of Pediatrics, Division of Pediatric Allergy and Immunology, Duke University Medical Center, Durham, NC USA; 50 Department of Pediatrics, University of California, San Francisco School of Medicine, San Francisco, CA USA; 51 Academia de Ciência e Tecnologia, Sao Jose do Rio Preto, Brazil; 52 Department of Pediatrics, Division of Hematology-Oncology, University of Rochester Medical Center, Rochester, NY USA; 53 Faculty of Médecine, University Tunis El Manar, Tunis, Tunisia; 54 Department of Pediatrics: Immuno-Hematology and Stem Cell Transplantation, Bone Marrow Transplantation Center of Tunisia, Tunis, Tunisia; 55 Department of Pediatrics, Kurume University School of Medicine, 67 Asahi-machi, Kurume, Fukuoka, Japan; 56 Section of Allergy and Immunology, Rush University Medical Center, Chicago, IL USA; 57 Research Department, Belarusian Research Center for Pediatric Oncology, Hematology and Immunology, Minsk, Belarus; 58 Department of Pediatrics, Medizinische Fakultät Carl Gustav Carus, Technische Universität Dresden, Dresden, Germany; 59 Department of Pediatrics, Section of Immunology, Allergy and Rheumatology, Baylor College of Medicine and Texas Children’s Hospital, Houston, TX USA; 60 Department of Internal Medicine I, University Hospital Carl Gustav Carus, Dresden, Germany; 61 Medizinische Klinik und Poliklinik I, Universitätsklinikum Dresden, Dresden, Germany; 62 Center for Pediatrics and Adolescent Medicine, Medical Center, Faculty of Medicine, University of Freiburg, Freiburg, Germany; 63 Department of Pediatrics, University of Rochester School of Medicine and Dentistry, University of Rochester Golisano Children’s Hospital, Rochester, NY USA; 64 Sabah Women and Children’s Hospital, Sabah, Malaysia; 65 National Clinical Research Center for Child Health and disorders, Children Hospital of Chongqing Medical University, Chongqing, 400014 People’s Republic of China; 66 Belfast Health and Social Care Trust, Belfast, Northern Ireland, UK; 67 Regional Immunology Service, Belfast Health and Social Care Trust, Belfast, Northern Ireland, UK; 68 Department of Microbiology, Tumor and Cell Biology, Karolinska Institute, Stockholm, Sweden; 69 Department of Immunobiology, Yale University School of Medicine, Yale University, New Haven, CT USA; 70 X4 Pharmaceuticals, Inc, Cambridge, MA USA; 71 Department of Population Medicine, Harvard Medical School and Harvard Pilgrim Health Care Institute, Boston, MA USA; 72 Division of Allergy and Immunology, Massachusetts General Hospital for Children, Boston, MA USA

**Keywords:** CXCR4, myelokathexis, warts, autoimmunity, neutropenia, lymphopenia

## Abstract

Warts, hypogammaglobulinemia, infections, and myelokathexis (WHIM) syndrome (WS) is a combined immunodeficiency caused by gain-of-function mutations in the C-X-C chemokine receptor type 4 (*CXCR4*) gene. We characterize a unique international cohort of 66 patients, including 57 (86%) cases previously unreported, with variable clinical phenotypes. Of 17 distinct *CXCR4* genetic variants within our cohort, 11 were novel pathogenic variants affecting 15 individuals (23%). All variants affect the same CXCR4 region and impair CXCR4 internalization resulting in hyperactive signaling. The median age of diagnosis in our cohort (5.5 years) indicates WHIM syndrome can commonly present in childhood, although some patients are not diagnosed until adulthood. The prevalence and mean age of recognition and/or onset of clinical manifestations within our cohort were infections 88%/1.6 years, neutropenia 98%/3.8 years, lymphopenia 88%/5.0 years, and warts 40%/12.1 years. However, we report greater prevalence and variety of autoimmune complications of WHIM syndrome (21.2%) than reported previously. Patients with versus without family history of WHIM syndrome were diagnosed earlier (22%, average age 1.3 years versus 78%, average age 5 years, respectively). Patients with a family history of WHIM syndrome also received earlier treatment, experienced less hospitalization, and had less end-organ damage. This observation reinforces previous reports that early treatment for WHIM syndrome improves outcomes. Only one patient died; death was attributed to complications of hematopoietic stem cell transplantation. The variable expressivity of WHIM syndrome in pediatric patients delays their diagnosis and therapy. Early-onset bacterial infections with severe neutropenia and/or lymphopenia should prompt genetic testing for WHIM syndrome, even in the absence of warts.

**Supplementary Information:**

The online version contains supplementary material available at 10.1007/s10875-022-01312-7.

## Introduction

WHIM syndrome (warts, hypogammaglobulinemia, infections, myelokathexis) is a rare multi-system combined immunodeficiency most often caused by autosomal dominant pathogenic variants in the *CXCR4* gene region coding for the C-terminus of the C-X-C chemokine receptor type 4. In selected cases, variants in *CXCR2* have been reported while other cases are unresolved since no genetic abnormalities were found yet the patients presented clinically with WHIM syndrome [1–4]. In cases of WHIM syndrome with *CXCR4* gain-of-function (GOF) variants, CXCR4 internalization is decreased, thus prolonging the interaction of CXCR4 and its ligand CXCL12, leading to hyperactive signaling. Myeloid and lymphoid cells fail to response to CXCL12 gradients and to egress from the bone marrow into periphery and/or to sites of inflammation [2, 5, 6]. Hence, neutrophils overmature in the bone marrow and cause a distinct pathological entity coined “myelokathexis” (Gr. *marrow retention*) [7–9]. Bone marrow pathology in WHIM syndrome is not limited to neutrophils, but also shapes B and T cell development and homing. As an example of its pleiotropic function, CXCR4 also plays a role in development of the cardiac system, as well as in cancer. CXCR4 overexpression contributes to tumor growth, invasion, angiogenesis, metastasis, relapse, and therapeutic resistance [10–13]. The wide spectrum of multi-system clinical presentations, often with incomplete penetrance and expressivity, together with rareness and lack of awareness of the disease, relative infrequency of life-threatening infection and incorrect interpretation of bone marrow pathology poses a significant diagnostic challenge even among members of the same family with identical heterozygous *CXCR4* pathogenic variants [14].

Since the first description of myelokathexis in 1964 and identification of the molecular basis of WHIM syndrome in 2003, more than 100 cases of WHIM have been reported in the literature based on individual case reports and patient registries [2, 7, 8]. Most patients have pan-leukopenia, including neutropenia and lymphopenia, and associated hypogammaglobulinemia. Registry-based studies of WHIM syndrome have described wide heterogeneity in the clinical phenotypes, including variable incidence of recurrent infections (including respiratory tract infections, skin infections, periodontal disease, osteomyelitis, meningitis), susceptibility to human papilloma virus (HPV), conotruncal heart defects, and an increased risk of malignancy, especially EBV and HPV-associated lymphoproliferative diseases [10, 15–18]. However, there are fewer data on the natural history of manifestations of the disease in childhood, and on the implications of delayed diagnosis for the risk of end-organ damage. Disease complications such as bronchiectasis, hearing loss, and cancer may be prevented or better managed with early diagnosis and intervention [10]. This is especially critical in the era of precision medicine, where targeted therapy antagonizing CXCR4 signaling is being studied and developed [19, 20].

Despite highly variable clinical presentations, historically, WHIM syndrome has been a clinicopathologic diagnosis. Bone marrow biopsy reveals the hallmark feature of myelokathexis [21]. Neutropenia, a laboratory hallmark of WHIM, arises early on (often around birth); however, it normalizes intermittently with infections, and therefore could be unnoticed and may delay evaluation [22, 23]. Although warts develop in 61% of cases, this clinical sign may be absent early in life and further hinders the diagnosis of WHIM syndrome [10]. As genetic testing has become more readily available, sequencing for pathogenic gain-of-function variants in the C terminal region of the *CXCR4* gene is often used to confirm a clinical suspicion of WHIM syndrome. The advantage of genetic testing is that it is non-invasive and easily accessible. Therefore, it may facilitate early diagnosis and serve as an alternative to diagnosis by bone marrow biopsy to confirm myelokathexis.

The goal of our study was to characterize the disease progression and clinical spectrum of WHIM syndrome across the life span of patients, with a focus on identifying clinical presentations prominent in early childhood that can facilitate timely recognition of the disease. Furthermore, we sought to compare clinical outcomes of those identified by family history versus clinical presentation of sporadic cases with a de novo variant, and to evaluate the importance of early diagnosis on disease outcomes.

## Material and Methods

A retrospective chart review was conducted of an international cohort of patients with WHIM syndrome from 42 centers in 21 countries across Africa, Asia, Australia/Oceania, Europe, North America, and South America. Centers were invited to participate through outreach for collaboration with international, regional-federal, or national immunological societies. Inclusion criteria were based on adapted ESID-PAGID diagnostic criteria for WHIM syndrome: (1) definitive WHIM syndrome diagnosis—chronic (non-cyclic) neutropenia (ANC less than 1000/μL) and a mutation in the intracellular C-tail of *CXCR4*; (2) probable WHIM syndrome diagnosis—chronic neutropenia (ANC less than 500/μL), myelokathexis, and at least two of the following criteria, chronic or recurrent warts, chronic lymphopenia (ALC less than 1500/μL), hypogammaglobulinemia, and/or a parent with neutropenia and warts.

Age at diagnosis was defined as age when patients fulfilled the adapted ESID-PAGID diagnostic criteria for WHIM syndrome. Patients were characterized (1) as prospectively diagnosed by genetic testing, which was prompted by a positive family history (FH) and diagnosis below the age 1 year; (2) as diagnosed by genetic testing prompted by a known WHIM syndrome diagnosis of a relative, which triggered evaluation for WHIM, regardless of age or degree of kinship; (3) as diagnosed based on clinical signs (Sx) of WHIM syndrome, which triggered evaluation for primary immunodeficiency and subsequent diagnosis of WHIM syndrome. Initial WHIM-related manifestation was defined as the first presenting sign or symptom of WHIM syndrome recorded in the medical record that contributed to a retrospective diagnosis based on chart review, as advocated by Prince and Berman in 2012 [24]. Disease progression was calculated as, number of patients with a specific disease manifestation divided by the number of patients tested at a specific age.

A structured datasheet was utilized to collect clinical information of 66 patients with WHIM syndrome from their treating physicians, including the following: patient sex, age (as of December 2020), age at diagnosis, race and ethnicity, genotype (specific CXCR4 variants), diagnostic details, age at onset of neutropenia, lymphopenia, hypogammaglobulinemia, presence of myelokathexis, infectious complications, end-organ damage, presence of malignancy, heart defects, malformations or autoimmunity, and therapies trialed (including response and complications). Of the study subjects, 9 cases had been previously described in published case reports [14, 21, 25–31]. As a separate initiative, we included and analyzed 2 patients with autoimmune manifestations from Australia and the USA: these patients added to our understanding of the autoimmune (AI) manifestations in WHIM syndrome but, due to lack of sufficient data were not included in the calculation of the prevalence of these manifestations. Immune thrombocytopenic purpura (ITP) was defined according to the EHASWGT as isolated thrombocytopenia (< 100 × 10^9^/L) in the absence of other causes [32]. Autoimmune hemolytic anemia (AIHA) was defined as hemolytic anemia with positive direct antiglobulin test (DAT) [33].

Therapeutic response was scored based on clinical judgment by treating physicians for all annotated cases using the following criteria: “non” = no clinical response to the intervention was seen or side effects were limiting; “partial” = some clinical improvement to the intervention was seen but therapeutic escalation was ultimately required for stabilization—specifically (1) G-CSF improved ANC counts but no effect on susceptibility to infections and (2) IgGRT and antibiotics prophylaxis improved infectious burden by < 50%; or “full” = clinical improvement to the intervention was seen and no subsequent escalation has been required for stabilization to date—specifically (1) G-CSF improved ANC counts and improved susceptibility to infections and (2) IgGRT and antibiotics prophylaxis improved infectious burden by > 50%. Infectious burden was assessed by treating physician.

Statistical comparisons of categorical variables were performed using Fisher’s exact test. To evaluate continuous variables, t-statistic and Kruskal–Wallis tests were applied. All reported *p* values are 2-sided, and *p* values less than 0.05 were considered significant. The R statistical software (version R 3.6.2) was used to perform all statistical analyses.

### Genetics

*CXCR4* sequencing was performed using standard techniques (whole-exome, panel-based, Sanger sequencing) in local laboratories, and referenced to NM_003467.3 and NP_003458.1.

All observed *CXCR4* variants were classified according to the 2015 standards and guidelines for the interpretation of sequence variants established by the American College of Medical Genetics and Genomics (ACMG) and the Association for Molecular Pathology (AMP) [34]. Definitions of criteria for classifying pathogenic variants are published by Richards and colleagues [34]. Additional general guidance developed by the Clinical Genome Resource (ClinGen) for the PVS1[35] and PS3[36] criteria were also incorporated. Of note, the PVS1 criterion is applied only to loss of function variants; given that the disease mechanism of WHIM syndrome is gain of function, PVS1 was not applied to any *CXCR4* variants. In lieu of PVS1, the PM4 criterion was applied to variants resulting in protein length changes. Although this criterion is typically applied to in-frame deletions/insertions, ClinGen recommendations (https://clinicalgenome.org/docs/variant-curation-standard-operating-procedure-version-2/) suggest PM4 can also potentially be applied to variants that cause premature truncation, but not nonsense-mediated decay, such as those observed in the *CXCR4* variants reported in association with WHIM syndrome that lead to production of C-terminally truncated receptors lacking residues critical for receptor internalization. Although the in silico predictor CADD [37, 38] suggests that the observed variants are likely to be damaging to protein function, the PP3 criterion was not applied to avoid double counting of evidence used to support the application of PM4. Additionally, the PP1 criterion used to support co-segregation of the variant within one or more families was applied if the genotype of the affected family member(s) was confirmed. The PS4 criterion (used at a moderate strength when a variant was observed in multiple unrelated patients with the same phenotype) was applied if the variant was observed in ≥ 3 unrelated individuals asserted to have WHIM syndrome. A specific phenotype sufficient for application of the PP4 criterion was defined as the presence of most features of WHIM, including myelokathexis.

### Internalization Assay

The cDNA of human CXCR4 (GenBank accession no NM_003467) carrying the consensus Kozak sequence at the 5′ end (GCCGCCACCatg) and 36 nucleotides of the 3′UTR at the 3′ end was synthesized at Genewiz (Leipzig, Germany) and cloned in pcDNA3.1 (Hygro+) via NheI/EcoRV to give pcDNA3.1_CXCR4. CXCR4_WHIM syndrome variants were generated by site-directed mutagenesis, using the QuickChange II XL kit (Agilent Technologies), the primers (listed in table below) and pcDNA3.1_CXCR4 as template. S346Pfs*12 was synthesized at Genewiz and cloned in pcDNA3.1 (Hygro+) via NheI/EcoRV. Plasmids were generated using the Endofree Plasmid MaxiPrep Kit (Qiagen) and all plasmid preparations were sequenced (Microsynth, CMV forward, BGH reverse) (STable3).

K562 (CCL-243, ATCC) cells were cultured in IMDM (Gibco) containing 10% FCS (Sigma) and penicillin-streptomycin (Gibco). Starvation was performed in IMDM, 0.5% BSA (Sigma), penicillin-streptomycin overnight.

K562 cells (2.5 × 10^6^ cells in 250 μl/cuvette) were transiently transfected by electroporation with 25 μg pcDNA3.1_CXCR4 plasmids per cuvette in Ingenio Solution (Mirus), using a Gene pulser Xcell device (BioRad) and exponential decay protocol (250 V, 1000 μF) in 0.4 cm cuvettes followed by immediate transfer to 6-well plates containing 2.25 ml pre-equilibrated culture media. After 24 h, transfected cells (1 × 10^5^ cell/well) were seeded in 96-well plates and serum-starved overnight. Cells were then resuspended in warm incubation buffer (HBSS with Ca^2+^ and Mg^2+^ + 0.5% BSA + 20 mM Hepes pH 7.4) and stimulated with CXCL12 for 45 min or 4 h at 37 °C, 5% CO_2_. After incubation, the cells were washed twice with cold incubation buffer and then stained with CXCR4 12G5-APC antibody (BD, 1:20 dilution in incubation buffer) for 20 min at 4 °C. After washing and resuspending in flow buffer (HBSS with Ca^2+^ and Mg^2+^+ 0.1% BSA + 20 mM Hepes pH 7.4), the samples were measured on Cytoflex (Beckman-Coulter) and analyzed in FCS Express software (De Novo Software). Cells were gated based on the FSC/SSC and isotype control and the mean fluorescence intensity (MFI) of CXCR4+ population was used in subsequent analysis.

Statistical analysis was performed in the GraphPad Prism software. *p* Values < 0.05 were considered statistically significant and set as follows: *p < 0.05; **p < 0.01; ***p < 0.001; ^ns^*p* ≥ 0.05. Unpaired 2-tailed *t* test was used. The number of independent experiments (*n*) is stated in each figure legend.

## Results

### Demographic Characterization

The study cohort comprises 66 patients with WHIM syndrome, diagnosed according to the adapted ESID-PAGID guidelines: the majority met criteria for a definite diagnosis (64), and two cases met criteria for a probable WHIM diagnosis. Our cohort includes 57 unique unreported cases of WHIM syndrome, those reported previously were individual case reports (*n* = 9) referenced in Supplementary Table 1. Our cohort does not have major overlaps with any prior reports from academic centers with high expertise in Europe (Italy, France) or the National Institutes of Health in the USA. Table 1 summarizes the demographics of the subjects. Our cohort includes nearly twice as many children (*n* = 43, < 18 years old) as adults (*n* = 23, > 18 years old). The median age at diagnosis was 5.5 (range 2 weeks–51 years). There was a predominance of female patients (62% female, *n* = 41; 38% male, *n* = 25). The diagnosis of the majority of patients (79%, *n* = 52) was prompted by clinical signs (Sx) related to WHIM, rather than via positive family history (FH, 20%, *n* = 13) and/or newborn screening for SCID (NBS-SCID, 1.5%, *n* = 1) with low T cell receptor excision circles (TRECs). In 71% (*n* = 47) of patients, both genetic testing and diagnostic bone marrow biopsy were used to confirm WHIM diagnosis. In 26% (*n* = 17) of patients, diagnosis of WHIM syndrome was confirmed solely based on genetic testing, and the remaining 2 patients were diagnosed by bone marrow biopsy and clinical signs only. Only 22.7% (*n* = 15) of patients in our cohort presented with all four features of the WHIM acronym. An asterisk in parenthesis (*) indicates that a bone marrow biopsy was not performed but leukopenia was present. The most common phenotype combination was HIM with 24% (*n* = 16), followed by IM in 15.2% (*n* = 10), I(*) 7.6% (*n* = 5), WI(*) 7.6% (*n* = 5), HI(*) 6.1% (*n* = 4), WIM 4.5% (*n* = 3), HM 4.5% (*n* = 3), WHI(*) 4.5% (*n* = 3), M or (*) 1.5% (*n* = 1) each (Supplementary Table 1).

**Table 1:** Demographic characterization of study subjects

Attribute	(*n* = 66)
Sex, *n* (%)	
Male	25 (37.9)
Female	41 (62.1)
Race	
Non-Hispanic White	46 (69.7)
Hispanic White	2 (3.0)
African American	2 (3.0)
Asian/Pacific Islander	13 (19.7)
Others/unknown	3 (4,5)
Geography	
Northern America	17 (25.8)
South America	3 (4.5)
Northern Europe	3 (4.5)
Western Europe	9 (13.6)
Southern Europe	1 (1.5)
Eastern Europe	17 (25.8)
Northern Africa	1 (1.5)
Western Asia	2 (3.0)
Eastern Asia	11 (16.7)
Southeast Asia	1 (1.5)
Australia and New Zealand	1 (1.5)
Age, median	15.0
Age at diagnosis, median	5.5
Mutation, *n* (%)	
De novo	39 (59.1)
Familial	20 (30.3)
Unknown	7 (10.6)
Confirmatory diagnostic, n (%)	
Genetic alone	17 (25.8)
Myelokathexis alone	2 (3.0)
Both	47 (71.1)
Age at clinical presentations, mean (SD)	
Infections	1.6 (2.7)
Neutropenia	3.5 (5.4)
Lymphopenia	5.3 (6.0)
Hypogammaglobulinemia	7.3 (8.5)
Warts	12.0 (8.7)

### Genetic Characterizations

**Fig. 1:**
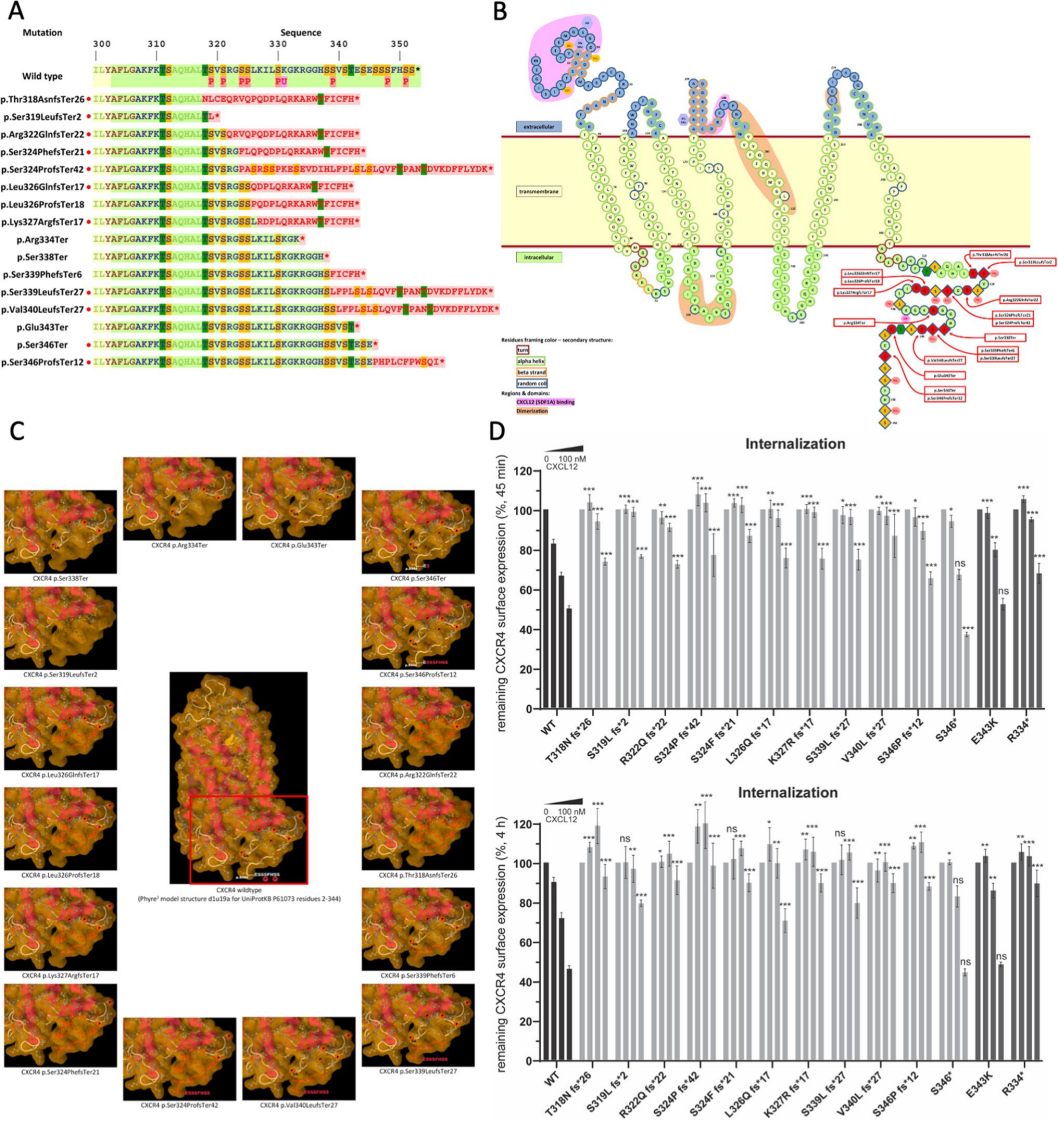
Genetic characterization of 66 patients with WHIM syndrome. **A** CXCR4 full-length sequence of the native CXCR4 aligned with CXCR4 mutations in C-terminus identified in WHIM syndrome patients. Novel mutations are highlighted with a red dot. **B** Snake plot of the schematic structure of CXCR4 with mutations highlighted in red. Light pink … CXCL12 (SDF1A) binding domain, yellow … dimerization domain. **C** Phyre^2^ three-dimensional model structure of the chemokine receptor CXCR4 residues 2–334, with conformational change of CXCR4 truncating mutations highlighted in red. **D** K562 cells transfected with WT CXCR4 and the indicated variants were stimulated with CXCL12 (vehicle, 1 nM, 10 nM, 100 nM) for 45 min and 4 h, respectively, and the surface expression of CXCR4 was measured by flow cytometry. Values are expressed as % remaining CXCR4 signal compared to vehicle-treated cells. Values represent mean ± SEM, *n* = 3–12)

Of 66 patients, 64 cases were reported to have variants in the C-terminal domain of *CXCR4*. In one case, genetic information was lost, and in 2 patients, the diagnosis of WHIM syndrome was based on clinical presentation and bone marrow morphology, according to ESID-PAGID diagnostic criteria for WHIM syndrome. We identified 17 distinct variants in our cohort, resulting in 16 different amino acid changes (Fig. 1A). Four variants were nonsense-truncating variants and 12 were frameshift variants, all positioned (p.318 to p.346) in the intracellular region of the C terminus (p.303 to p.352), similar to prior reports [5] (Fig. 1B, C). Nonsense mutations were reported in 45 patients (68%) and frameshift mutations in 17 patients (26%). In 38 (64%) cases, mutations occurred de novo, while the other 21 cases were distributed among 15 pedigrees (SFigure1). Eleven of seventeen variants (64%) are previously unpublished in WHIM patients and represent 23% of our cohort (15 cases). As expected, p.R334* (47%, *n* = 31), and p.S338* (17%, *n* = 11) were the most common variants in our cohort. We identified two different genotypes (c.1013C>G and c.1013C>A) resulting in p.S338*, with the c.1013C>A (1/11, 9%) never reported in WHIM syndrome. Both variants create a premature stop codon, tCa>tGa, and tCa>tAa, respectively, thus truncating the C terminus. The 14 remaining amino acid changes were distributed among the remaining genotyped cases in only 1 to 4 cases, each (STable2). We observed a marked phenotypic heterogeneity in patients with the same genotype, even within the same family, with no obvious correlation between genotype and clinical manifestations (SFigure1). All 17 distinct variants in our study cohort, including the 11 novel variants, are predicted to be deleterious with a CADD PHRED value above 15. (range: 26.5 to 39) (STable2). All variants observed in the cohort were classified according to the ACMG/AMP 2015 standards and guidelines for the interpretation of sequence variants [34]. Of the 17 identified variants, 15 were classified as likely pathogenic or pathogenic (STable2). These classifications were primarily supported by the location of the variants in the critical C-terminus, absence in the gnomAD population database [39], availability of inheritance information gleaned from family studies (variant co-segregation with disease or variant de novo status), and presence of WHIM-specific phenotypic features observed in affected individuals.

The receptor desensitization defect is believed to be a hallmark of *CXCR4* mutations found in WHIM syndrome patients and is the mechanism responsible for their gain-of-function phenotype [40]. To confirm that the newly identified *CXCR4* variants (T318N fs*26, S319Lfs*2, R322Qfs*22, S324Ffs*21, S324Pfs*42, L326Q fs*17, K327R fs*17, S339L fs*27, V340L fs*27, S346* and S346Pfs*12) also displayed the same defects, we measured their internalization responses to the natural ligand CXCL12. *CXCR4-WT, CXCR4-WHIM* (R334* and E343K) and the novel CXCR4 variants were expressed in K562 cell line (lacking endogenous CXCR4 expression [41]), stimulated with increasing concentrations of ligand and stained for remaining CXCR4 expression on the cell surface (Fig. 1D). As a result of CXCL12 stimulation, CXCR4-WT receptor surface levels decreased in a dose-dependent manner. CXCR4-R334*, the most frequent WHIM syndrome variant, displayed a significant internalization defect at all CXCL12 concentrations used, while E343K, a missense variant found in WHIM syndrome patients [42], altered the internalization response only at lower ligand concentrations. All investigated new variants showed defective CXCR4 internalization. The S346* variant was the least affected, displaying significant impairment only at 1nM CXCL12. Interestingly, while a subset of this patient’s neutrophils demonstrates the typical myelokathexis changes with long internuclear strands, the majority of neutrophils had a normal morphology.

In addition to in silico and functional testing, we conducted a database search to find identical or similar (different mutation on cDNA level with identical amino acid sequence) somatic *CXCR4* gain-of-function mutations in malignant diseases. Conditions included Waldenström’s macroglobulinemia, lymphoma, and IgM monoclonal gammopathy. All literature reported malignant variants could be identified as somatic *CXCR4* gain-of-function mutations. Of the 11 novel variants, 6 variants possessed identical somatic nucleotide changes and 5 variants resulted in identical amino acid sequences but different nucleotide changes (STable2).

### Initial Clinical Manifestation of WHIM Syndrome

**Fig. 2:**
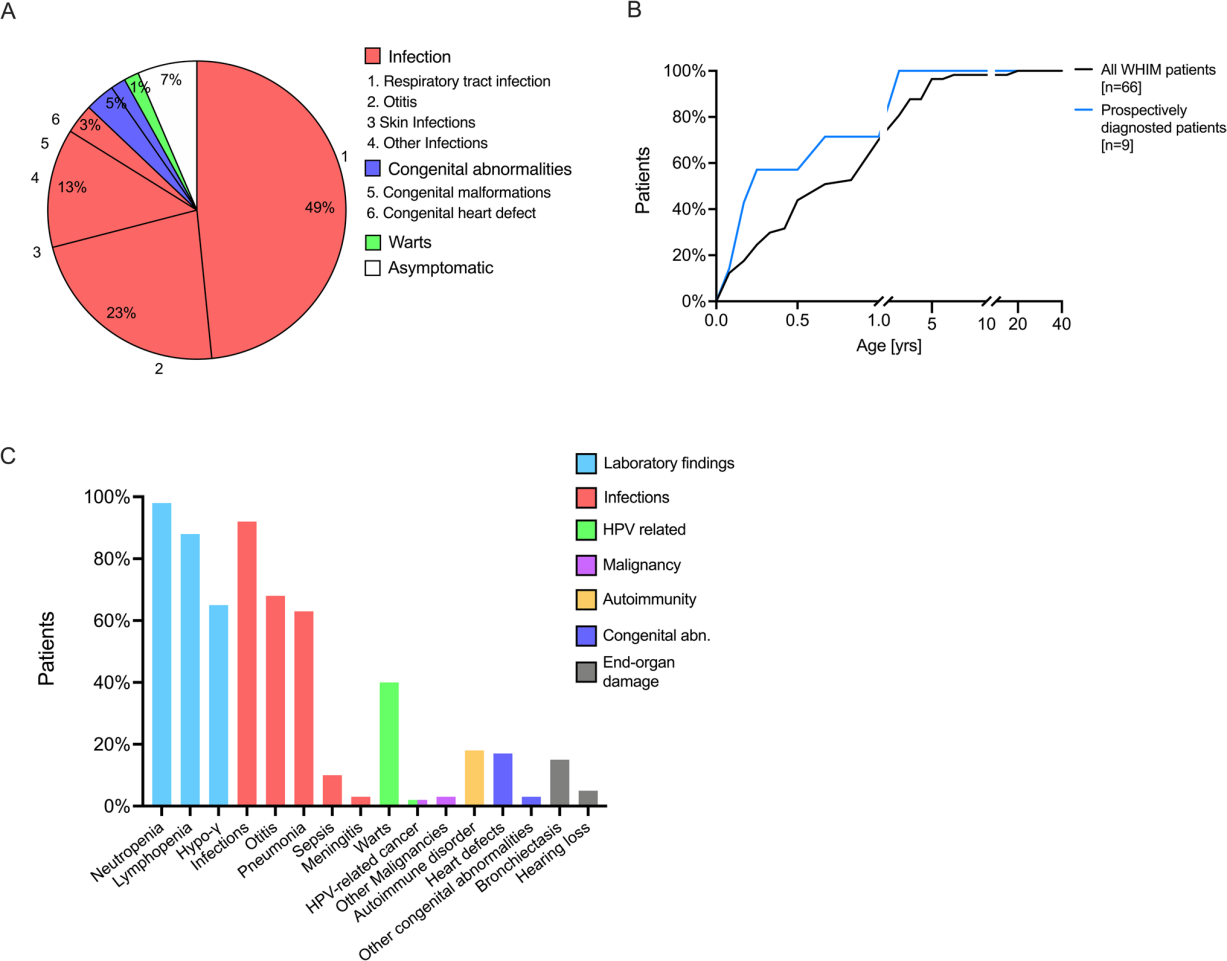
Analysis of WHIM syndrome-related clinical manifestation and laboratory findings. **A** Type of first WHIM syndrome-related clinical manifestation. **B** Cumulative percentage of patients suffering from their first WHIM syndrome-related clinical manifestation at a given age for all patients (black line, *n* = 66), and prospectively diagnosed patients based on positive family history and < 1 year of age (blue line, *n* = 9). **C** Lifetime prevalence of individual WHIM syndrome-related manifestations and laboratory findings (frequency as % total cases)

First, we assessed the initial type of WHIM-related illnesses that occurred within the first year of clinical presentation. Infections were the most common initial clinical manifestation of WHIM syndrome (88%), dominated by upper and lower respiratory tract infection (49%), followed by otitis media (23%), and skin infections (13%), while HPV-related manifestations and warts were uncommon (2%). Five percent of patients initially presented with congenital abnormalities, including malformation (3%, maxillofacial defects including orofacial cleft) and heart defects (2%, patent ductus arteriosus) (Fig. 2A). Seventy percent of symptomatic patients had their first initial WHIM clinical manifestation before the age of 1 year, and 96% had it before the age of 5 years. To account for any retrospective, observational bias, we calculated the age at initial clinical manifestation of those WHIM syndrome patients that were prospectively diagnosed based on positive family history and age < 1 year (*n* = 9). Over 50% of those prospectively diagnosed patients had their first WHIM-related manifestation before the age of 3 months and over 90% of prospectively diagnosed patients, had their first WHIM-related manifestation before the age before 1 year of age (Fig. 2B).

## WHIM Clinical Manifestations Are Diverse

The majority of patients (98%) had laboratory abnormalities, with non-cyclic neutropenia (98%) being the most prevalent finding, followed by lymphopenia (88%) and hypogammaglobulinemia (65%). Infections were seen in 92% of patients, with otitis media occurring in 68% and pneumonia in 63% of patients. Bacterial meningitis and bacteremia resulting in sepsis had a cumulative lifetime prevalence of 13% in our cohort. Infection-associated end-organ damage, including bronchiectasis and bronchiolectasis due to recurrent pneumonias, and hearing loss due to recurrent otitis, were seen in 20% of patients. HPV-related manifestations were seen in 42% of patients, with warts occurring variably on face, hands, feet, arms, or legs in 40% of patients. No cases of HPV-related malignancy were reported in this cohort. However, two patients developed non-HPV-related malignancy: one adult patient with basal cell cancer and an infant with melanotic neuroectodermal tumor. Autoimmune (AI) complications were observed in 21% of patients. Heart defects were observed in 17% of patients, including patent ductus arteriosus, tetralogy of Fallot, right-sided aortic arch, tricuspid valve insufficiency, aortic valve insufficiency, and Wolff–Parkinson–White (WPW) syndrome. In addition, minor malformations including maxillofacial anomalies were seen in 3% (Fig. 2C).

### Disease Progression of WHIM Syndrome

**Fig. 3:**
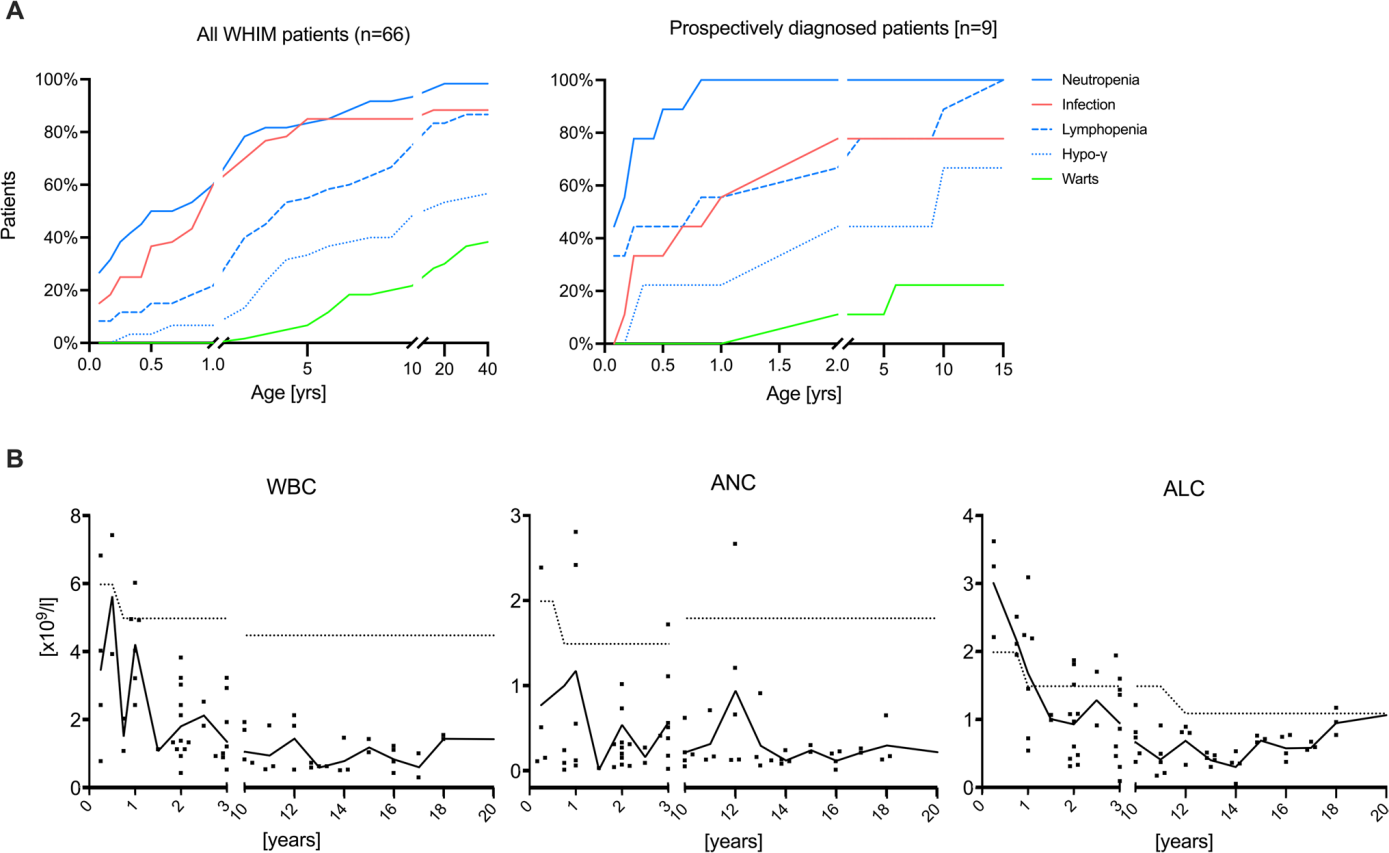
Clinical and immunological disease progression of WHIM syndrome. **A** Cumulative percentage of WHIM syndrome-related manifestations at a given age in all WHIM syndrome patients (*n* = 66, left panel) and patients prospectively diagnosed patients based on positive family history and < 1 year of age (*n* = 9, right panel). **B** Longitudinal analysis of WBC, ANC, and ALC counts (*n* = 35). Black line represents median value at a given age, dots represent individual patient’s values at a given time point and dotted line is age matched reference values

Clinical manifestations in our cohort (*n* = 66) varied across the life span; we therefore calculated the cumulative percentage of WHIM syndrome-related manifestations tested at a given age. STable1 displays the individual observational period, with a median of 12 years. The predominant clinical presentation in infancy was susceptibility to bacterial infections, more than 50% (*n* = 36) of them had documented bacterial infections during the first year of life. Overall, the most common infections were otitis media and pneumonia developing at a median age of 3 years. Neutropenia was diagnosed prior to 4 weeks of age in 26% of our study population (*n* = 66) and in 50% by the age of 6 months. Lymphopenia and hypogammaglobulinemia were diagnosed later in life, with more than half of the cohort being diagnosed at the age of 4 years (*n* = 34) and 10 years (*n* = 33), respectively. Three patients reported cutaneous warts during early childhood. The incidence of warts increased to more than 25% in adulthood, with a median reported onset of 14 years (*n* = 17) (Fig. 3A).

Patients that were prospectively diagnosed based on positive family history and < 1 year of age (*n* = 9), with a median observational period of 9.2 years, had a similar median onset of infections, with 50% developing their first infectious complication at 1 year of age. Neutropenia and lymphopenia were observed in 50% and 33% prior to 4 weeks of age, respectively. Neutropenia prevalence increased to 100% at age of 10 months, while lymphopenia was observed in 50% at the age of 10 months and increased to 80% at the age of 10 years. Hypogammaglobulinemia was rarely diagnosed in early life, with more than half of the cohort being diagnosed at the age 10 years. Warts were never reported in infancy, with one patient developing warts at age 2 years (11%) and 2 additional patients at age 6 and 9 years, respectively (22% and 33%). Patients presented a mean lowest value during follow-up of 852 ± 386 leukocytes/mm^3^ and 113 ± 95 neutrophils/mm^3^. In addition, we observed a mean lowest value during follow-up of ALC count of 526 ± 153 cells/mm^3^ and an AMC count of 51 ± 37 cells/mm^3^ (Supplementary Figure 2).

Furthermore, we performed a longitudinal analysis of patients’ laboratory values for those that neither received G-CSF or CXCR4 antagonists. Lab values were excluded if patients were actively treated for infection at this time point (*n* = 35, median observational period of 6.5 years). Patients with WHIM syndrome displayed moderate leukopenia prior to 4 weeks of age, with a median WBC count of 3920 cells/mm^3^ (*n* = 5), until age of 1 year with 3590 cells/mm^3^ (*n* = 8). However, with age, leukopenia progressively worsened with a median WBC count of 1350 cells/mm^3^ (*n* = 13) at age of 2 years, 1240 cells/mm^3^ (*n* = 13) at age of 5 years, and 830 cells/mm^3^ (*n* = 8) at age 10. Neutropenia varied through life; however, there was a trend towards a progressive loss of neutrophils with a median ANC count of 540 cells/mm^3^ (*n* = 6) around birth, to 1238 cells/mm^3^ (*n* = 8) at age 1 year, 547 cells/mm^3^ (*n* = 12) at age 2, and 197 cells/mm^3^ (*n* = 8) at age 5. The progressive loss was most noticeable with ALC: WHIM syndrome patients had overall normal lymphocyte counts at birth up to one year of age, with a median ALC of 2350 cells/mm^3^ (*n* = 4) and 1694 cells/mm^3^ (*n* = 6), respectively. However, we observed the development of a progressive lymphopenia in early childhood, with an ALC count of 939 cells/mm^3^ (*n* = 11) at age 2 and 787 cells/mm^3^ (*n* = 13) at age 5. Individual WBC, ANC, and ALC counts over time for every single patient can be seen in (Supplementary Figure 2). Analyzing individual patients longitudinally showed a progressive neutropenia in 20 patients (57%) and progressive lymphopenia in 25 patients (71%) (Fig. 3B).

### Treatment and End-Organ Damage

**Fig. 4:**
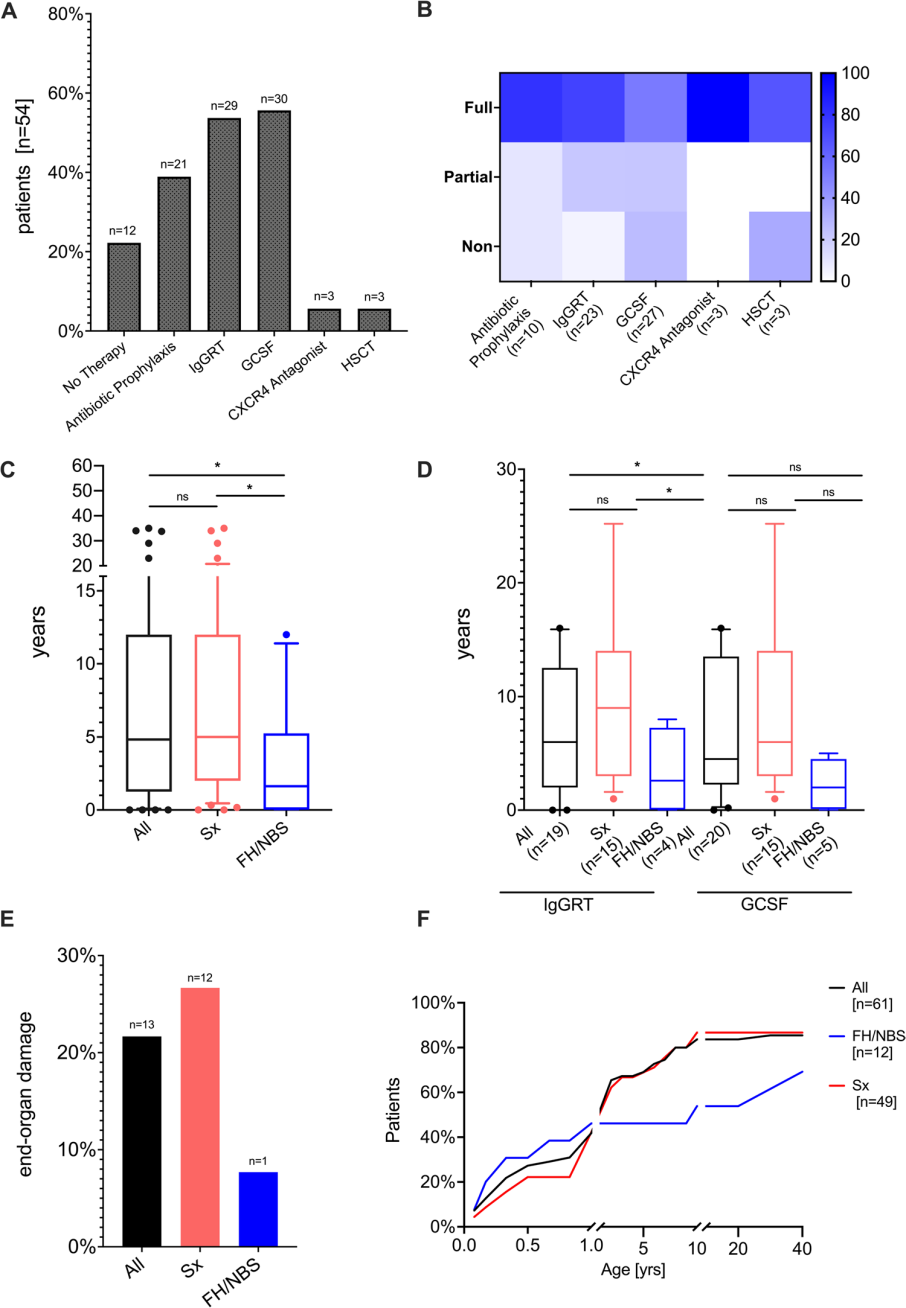
Treatment approaches and end-organ damage in WHIM syndrome. **A** Prevalence of individual treatment strategies. **B** Percent of patients with a treatment response that was scored using the following criteria: “non” = no clinical response or side effects were limiting, “partial” = clinical improvement but therapeutic escalation was required, or “full” = clinical improvement with no escalation. **C** Time from first record of neutropenia to final diagnosis of WHIM syndrome. All patients (black box plot), patients diagnosed by family history or newborn screening (FHS/NBS blue box plot) and patients diagnosed by clinical signs (Sx, red box plot). Whiskers-box plot represents (median ± quantile 0.1–0.9). **D** Time from first record of neutropenia to initiation of either GCSF or IgGRT. All patients (black box plot), patients diagnosed by family history or newborn screening (FHS/NBS blue box plot) and patients diagnosed by clinical signs (Sx, red box plot). Whiskers-box plot represents (median ± quantile 0.1–0.9). **E** Patients with end organ damage, including bronchiectasis and/or hearing loss of all patients (black bar), those diagnosed by clinical signs (red bar) and those diagnosed by family history (red bar). **F** Cumulative percentage of first hospital admission due to WHIM-related manifestation by age

Treatment data were available for 54 WHIM syndrome patients. Granulocyte colony-stimulating factor (G-CSF) was used in 55.7% (*n* = 30), IgG replacement therapy (IgGRT) in 53.7% (*n* = 29) and antibiotic prophylaxis in 38.9% (*n* = 23) to reduce susceptibility to infections (Fig. 4A). Therapeutic response was scored based on clinical judgment by treating physicians, for detailed definition see material and methods. G-CSF improved infectious complications and increased ANC counts in approximately 50% of patients, while 22% of patients showed improved ANC counts with no effect on susceptibility to infections and 26% of patients failed to increase ANC counts or ameliorate infection frequency in response to G-CSF treatment. Antibiotic prophylaxis and IgG replacement therapy were able to reduce infectious burden (> 50%) in 80% and 74% of patients, respectively, while partial response (< 50%) was observed in 10% and 22%, respectively. Only a minority of patients showed no response to antibiotic prophylaxis and IgGRT (10% and 4.35%), respectively (Fig. 4B). CXCR4 antagonist (plerixafor) was used in 5.6% (*n* = 3) of patients, which ameliorated the burden and frequency of infection and improved panleukopenia. Of patients, 5.6% (*n* = 3) underwent hematopoietic stem cell transplantation (HSCT) as definitive treatment for WHIM syndrome, with successful outcome in 2 patients; however, one patient died due to HSCT-associated complications. Details for the patients that underwent HSCT were described by Laberko et al. [43] Twenty-two percent of our cohort required no therapeutic intervention at the time of this study. Data on treatment response of different drug combinations were not considered in this analysis.

We observed a substantial diagnostic delay from first date of recorded neutropenia to the final molecular diagnosis of WHIM syndrome. FH and/or NBS played a crucial role in facilitating early recognition of WHIM syndrome: in 67% (*n* = 6/9) of subjects, diagnosis was prompted by FH or NBS within the first year of life with a median delay of 1.3 years; in comparison, in 14% (*n* = 7/49) of subjects, diagnosis was prompted by clinical signs within the first year of life with a median delay of 5 years (*p* = 0.0387) (Fig. 4C). Those patients with a diagnosis prompted by FH and/or NBS were more likely to receive early therapeutic interventions, e.g., G-CSF and IgGRT, with a median age of 2 and 2.6 years, respectively, compared to those with a diagnosis prompted by clinical signs with a median age of 6 and 9 years, respectively (IgGRT *p* = 0.04, G-CSF *p* = 0.09) (Fig. 4D).

Irreversible end-organ damage, including bronchiectasis and hearing loss, was significantly more common in those patients with a diagnosis prompted by clinical signs compared with those with a diagnosis prompted by FH/NBS. Of the former, 27% developed bronchiectasis (*n* = 9) and hearing loss (*n* = 3) secondary to infections, while only one patient in the FH/NBS group was diagnosed with bronchiectasis (8%, *p* = 0.03), and non-developed hearing loss. End-organ damage in WHIM appeared early in life with a median age of 9.6 years; however, we did not find a correlation of age and end-organ damage (*p* = 0.278) (Fig. 4E). Eighty-three percent of our cohort were admitted to the hospital due to WHIM-related complications, with a median first hospital admission at the age of 2 years. Patients with a family history of WHIM were less likely to be hospitalized compared to those whose diagnosis was prompted by clinical signs (69% vs 87%). We further observed that the age at which patients required inpatient treatment differed between those with a diagnosis prompted by FH/NBS and clinical signs, with a median age of 7 years and 2 years, respectively (Fig. 4F).

### Autoimmunity in WHIM Syndrome

**Fig. 5:**
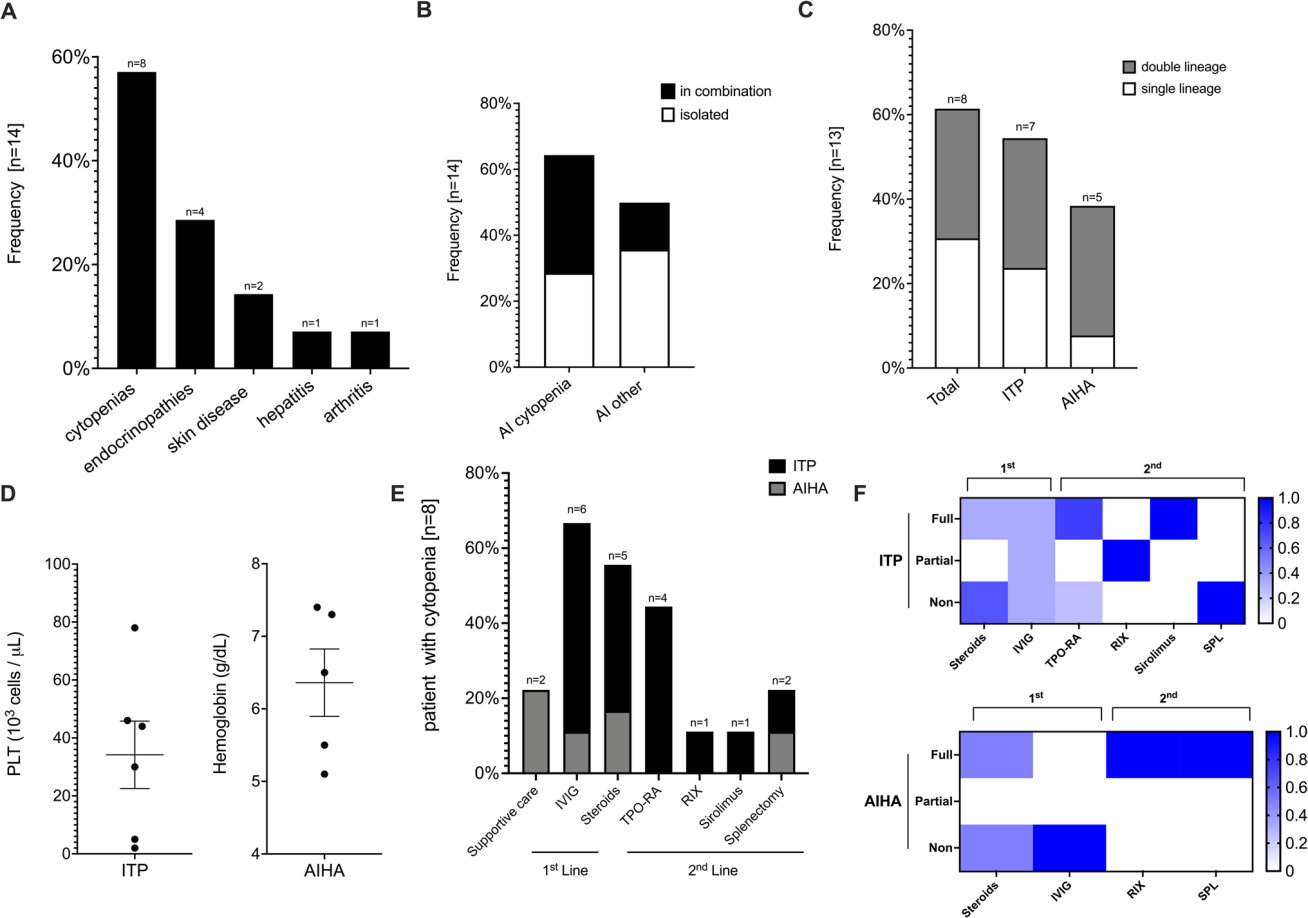
Autoimmunity and hyperinflammation in WHIM syndrome. **A** Prevalence of individual autoimmune (AI) and hyperinflammatory (HI) complications (frequency as % patients with subtype among all patients with AI or HI complications, *n* = 14). **B** Occurrence of autoimmune and hyperinflammatory complications in isolation or combination (frequency reported as above). **C** Prevalence of single- and multi-lineage cytopenias (frequency reported as above). **D** Platelet and hemoglobin nadir during flares (symbols representing individual values; median ± SEM shown). **E** Prevalence of individual treatment strategies used for ITP and AIHA. **F** Ratio of patients with a treatment response for first line (steroids ± IVIG) and second line (biologicals, immunosuppressives or splenectomy). Treatment response was scored using the following criteria: “non” = no clinical response or side effects were limiting, “partial” = clinical improvement but therapeutic escalation was required, or “full” = clinical improvement with no escalation

In our cohort of 66 patients with WHIM syndrome, we observed 12 patients (18%) with prominent autoimmune manifestations. In addition, we included and analyzed the cases of 2 patients with AI manifestations from Australia and the USA on the basis of a literature search and/or personal communication. Among patients with AI, we observed a predominance of female patients (71% females, 29% males). All WHIM syndrome patients with AI were non-Hispanic whites. AI manifestations were reported in patients that were significantly older than the overall cohort, with a median age of 22.1 years (range 6.2–47.2) compared with those without AI with a median age of 9.0 years (range 1.1–36.4). We could not identify any significant difference in lymphocyte counts, serum immunoglobulin levels or mutation between patients with and without AI. Taking into account the patients from this cohort and the literature search, the most frequent AI complications were autoimmune cytopenias (*n* = 8, 57.1%), followed by endocrinopathies (*n* = 4, 28.6%, type 1 diabetes [*n* = 3] and autoimmune thyroiditis [*n* = 1]), skin manifestations (*n* = 2, 14.3%, psoriasis [*n* = 1] and vitiligo [*n* = 1]) and two patients presented with arthritis and autoimmune hepatitis (each *n* = 1, 7.1%) (Fig. 5A). A total of 49.9% (7/14) of patients had more than one AI complication. Specifically, 35.7% of cytopenia cases presented with an additional autoimmune complication, while other autoimmune complications were more likely to occur in isolation (35.7%) (Fig. 5B). Immune thrombocytopenia purpura (ITP) was the most frequent autoimmune complication (*n* = 7, 53,8%), followed by Coombs-positive autoimmune hemolytic anemia (AIHA) (*n* = 5, 38%). Evans syndrome was observed in 4 patients (30.7%). No trilineage Evans syndrome was reported, with no autoimmune neutropenia observed in our cohort (Fig. 5C). Cytopenias had a nadir hemoglobin level of 6.4 g/dL attributed to AI (*n* = 5) and nadir platelet count of 37.000 cells/μL due to ITP (Fig. 5D).

### Treatment of Autoimmune Cytopenias in WHIM Syndrome

Treatment outcomes were reviewed in detail in patients with ITP (*n* = 7) and AIHA (*n* = 5). Mild AIHA (*n* = 2, median hemoglobin nadir 6.9 g/dL) required no specific therapy. High-dose intravenous immunoglobulin (IVIG) and steroids were frequently used as first-line therapy. Disease control using first-line therapy was achieved in a subset of patients (ITP 27%, AIHA 20%) only. Most patients with ITP required second-line therapy, which most frequently consisted of thrombopoietin receptor agonist (*n* = 1), B cell depletion with rituximab (*n* = 2), and mTOR inhibitor, sirolimus (*n* = 1). Second-line treatment of ITP fully or partially improved the outcome in the majority of cases (93%). Splenectomy seemed to be effective for controlling AIHA (*n* = 1) but failed to improve ITP (*n* = 1) (Fig. 5E, F).

## Discussion

Herein we describe 57 unreported WHIM syndrome cases, thereby expanding the number of published WHIM cases to 162 patients worldwide [5]. In general, our cohort showed similar rates of warts, hypogammaglobulinemia, infections, and neutropenia, while we observed fewer malignancies and deaths when compared with other cohorts [14, 20]. This is likely due the significantly younger median age (13.6 years) of our cohort compared to previously described cohorts with median ages of 22 and 29 years, respectively [14, 20]. In addition, the majority of our cohort patients were actively under treatment which likely confounds our study with a survival bias. In particular, the younger pediatric patients are likely a reflection of more accessible genetic screening, which has facilitated the diagnosis of pathogenic *CXCR4* variants even in patients with incomplete phenotypes. In the recently described review of 105 previously published patients, less than half (38%) fulfilled the diagnostic tetrad of WHIM syndrome. [5] Consistent with published data, we observed incomplete penetrance and variable severity in our cohort. Only 23% of the patients in our cohort presented with all four classic WHIM features. Lack of the diagnostic tetrad of WHIM syndrome correlated with age. Indeed, we observed that the majority of pediatric patients lacked the presence of HPV-related manifestations. Warts were reported in only a quarter of our cohort, and only 3 patients developed warts during early childhood. Our observations that warts developed at a median age of 14 years concurs with previous observations that the seroprevalence for HPV in the general population peaks during ages 15–30 years [44].

Hypogammaglobinemia was observed in two thirds of our cohort predominantly in late childhood and early adulthood. However, future research needs to determent the extent of antibody deficiency, e.g., vaccine response in patients with WHIM syndrome. As expected, neutropenia was the most prominent diagnostic feature in our pediatric cohort. However, neutropenia can remain unidentified during medical attention as neutrophil mobilization is induced during acute infections [23, 45]. Incomplete presentation of the pediatric disease course in combination with diagnostic challenges and lack of disease awareness contribute to diagnostic delays and worse outcomes [16, 46]. Indeed, when contrasting clinical outcomes of patients with early diagnosis due to family history to those diagnosed de novo, we observed a significant reduction in end-organ damage (bronchiectasis and hearing loss) as well as fewer hospital admissions. Sometimes diagnostic delay can be considerable. In one of the patients, the disorder had been labelled severe chronic neutropenia, and delayed the diagnosis until age 30 years, and in another, it was classified as combined immunodeficiency that delayed the diagnosis until age 46. In addition, end-organ damage, e.g., bronchiectasis, might be underreported in WHIM syndrome, as surveillance CT scans might be underutilized in WHIM syndrome.

Neutropenia was the earliest laboratory finding and the predominant, defining feature of WHIM syndrome throughout life. Only 26% of patients had neutropenia documented around birth and 50% had it by 6 months of age. The diagnosis of neutropenia can be delayed compared with the actual onset of a low neutrophil count due to a lack of awareness. Furthermore, in WHIM syndrome, ANC is not static and may normalize during infections [17]. Therefore, it is of great importance for ANC to be retested in patients who exhibit WHIM-like presentations, yet have anormal neutrophil count.

Interestingly, WHIM patients may come to medical attention at an asymptomatic stage, with low TREC levels at birth during NBS for SCID [26, 31]. Therefore, it may be of benefit to include a CBC with differential count and genetic testing for WHIM syndrome as the part of the evaluation process in case of abnormal NBS-SCID. The prevalence of WHIM syndrome is not known. In the French registry, it was estimated at about one in 4 million [5]. We have reported with Evans et al. that 3 of 6 infants with WHIM syndrome had low TRECs in the era of NBS-SCID [26, 47]. NBS-SCID screening may be an opportunity for early recognition of WHIM syndrome cases, even during clinically asymptomatic stages of the disease. An increased prevalence of WHIM syndrome would be predicted with more widespread screening. Consequently, early diagnosis could expedite therapeutic interventions, by using G-CSF to correct neutropenia, immunoglobulin (Ig) replacement to raise IgG levels, and prompt initiation of antibiotics to treat acute bacterial episodes or prevent infections [17]. Early therapeutic intervention with emerging CXCR4 antagonists, plerixafor and mavorixafor, is promising and could significantly mitigate morbidity and mortality by specifically targeting the mechanism of disease [19, 20]. However, additional evidence is needed to assess the therapeutic value of CXCR4 antagonists. Through our international cohort, we increased the list of CXCR4 pathogenic variants from 17 to 28 (STable4). Based on our cohort and prior reports, CXCR4 variants linked to WHIM syndrome are located in the region of p.318–346 [5]. Our report highlights the possibility of discovering new variants, especially in the era of NBS for SCID when patients with T cell lymphopenia may undergo sequencing around birth [47].

The majority of the newly identified CXCR4 variants showed defects in CXCL12-induced internalization comparable to R334*, the most frequent and most studied WHIM syndrome variant. This observation is in line with the requirement of intact phosphorylation motifs in the C-terminus for efficient CXCR4 internalization [48]. The S346* variant, which preserves one of the serine residues of the C-terminal GRK2/3 phosphorylation site [48], displayed only mild impairment in the internalization assays. Such mild internalization defects may be sufficient to cause a WHIM phenotype, as evidenced by patients harboring the E343K mutation [42]. Interestingly, the frameshift variant at the same position, S346Pfs*12, behaved differently and had defective internalization at all tested CXCL12 concentrations. The de novo additional sequence generated by the shift in the reading frame may thus confer previously unidentified inhibitory effects on receptor internalization that warrants further investigation.

A previously underappreciated observation from our cohort was a high prevalence of autoimmune complications in WHIM syndrome (18%). This is in line with immune dysregulation in other inborn errors of immunity leading to autoimmune and autoinflammatory complications in approximately 25% of patients with primary immunodeficiencies [49, 50]. In contrast, a recent analysis of the French national primary immunodeficiency registry does not report any autoimmune complications in patients with WHIM syndrome [51]. While the underlying mechanism of immune dysregulation in WHIM remains to be determined, abnormalities in T and B cell function and development that may result in impaired central and peripheral tolerance have been described. WHIM syndrome patients’ T cells form unstable immunological synapses with antigen-presenting cells (APCs), which may impair cytokine secretion and mitigate IgG class switching [52, 53]. In addition, a marked reduction in circulating B cells, restricted immunoglobulin heavy chain variable region diversity, and impaired class switching response have been described in WHIM [29, 46, 53–55]. Similar to observations in other combined immunodeficiencies, lymphopenia may contribute to the rescue of self-reactive T and B cell in the periphery, disrupting peripheral tolerance [56, 57]. In addition, aberrant CXCR4 expression has been shown to correlate with autoimmunity in lupus and CXCL12/CXCR4 signaling regulates T cell migration to target organs in Sjögren’s syndrome [58, 59]. In addition, mild thrombocytopenia of unknown etiology may also occur in untreated WHIM syndrome patients [5]. Further research is needed to understand the extent of impaired adaptive immunity given the possibility that B and T cell dysfunctions reported in patients with WHIM syndrome may play a role in immune dysregulation [15].

In conclusion, WHIM syndrome can present with variable clinical expressivity that results in diagnostic and therapeutic delays. Early-onset bacterial infections with severe pan-leukopenia should prompt early genetic testing for WHIM syndrome, even in the absence of warts. We expanded the clinical spectrum of WHIM syndrome to include autoimmune complications. Based on our data, we propose that workup for definitive diagnosis of WHIM should include genetic testing as a feasible alternative to bone marrow biopsy to assess myelokathexis, especially in young children. Therapeutic approach is versatile in WHIM syndrome and primarily based on clinical presentation and response. With targeted therapies on the horizon, laboratory immune biomarkers are needed for to determine the initiation and length of therapy with these novel treatment options.

## Supplementary Information

Supplementary Figure 1 (PNG 257 kb)

Supplementary Figure 2 (TIFF 5464 kb)

High Resolution Image (PNG 556 kb)

Supplementary Table 1 (XLSX 16 kb)

Supplementary Table 2 (XLSX 20 kb)

Supplementary Table 3 (DOCX 14 kb)

Supplementary Table 4 (XLSX 11 kb)

## Data Availability

The datasets generated and analyzed during the current study are available from the corresponding author upon reasonable request.
