# Supplementary figures and images for: Disease Progression of WHIM Syndrome in an International Cohort of 66 Pediatric and Adult Patients
Source: J Clin Immunol. 2022 Aug 10;42(8):1748–65. doi: 10.1007/s10875-022-01312-7 (PMC9700649; doi:10.1007/s10875-022-01312-7)

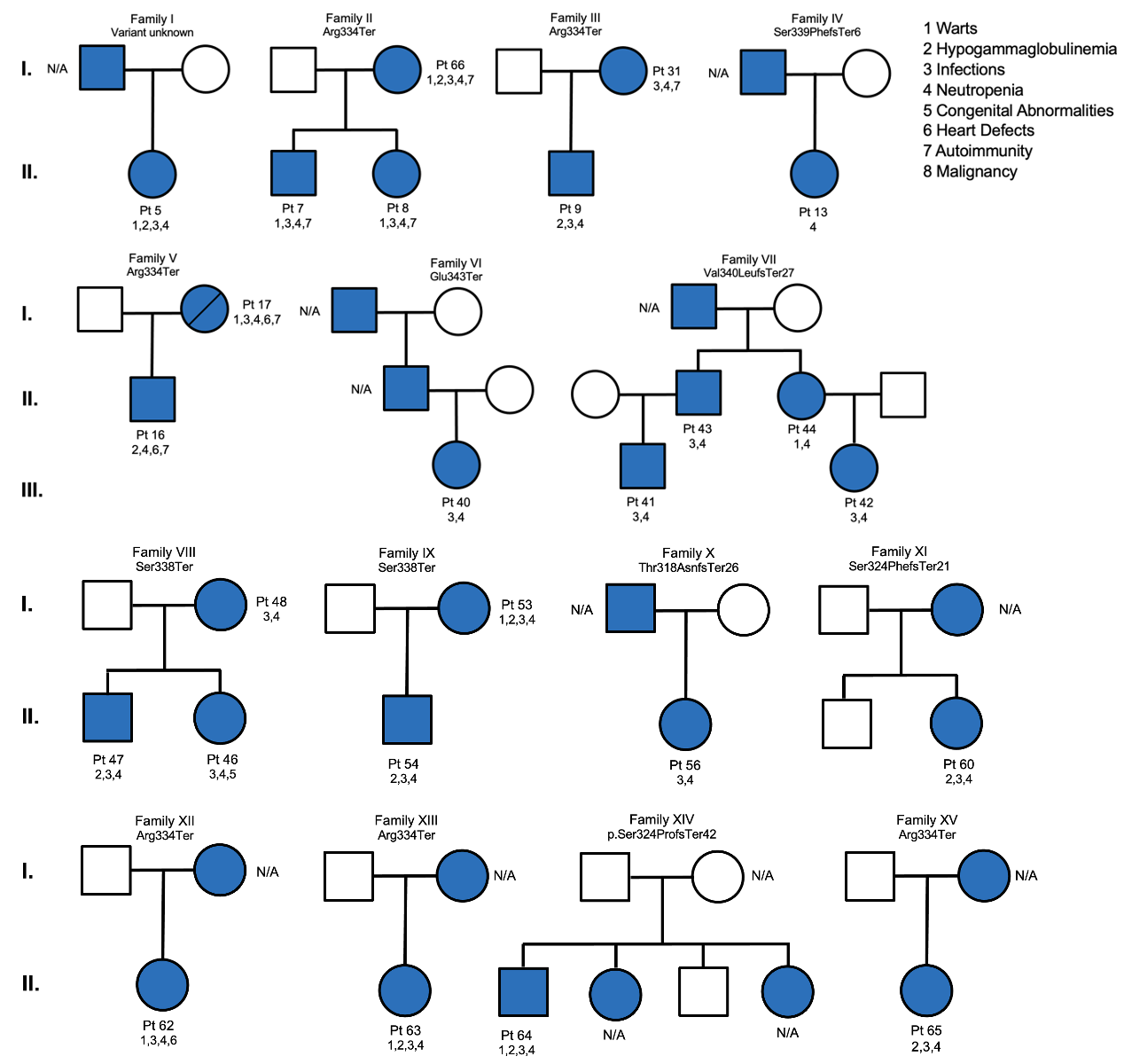

Supplement: Supplementary file 1 — (PNG 257 kb) [file 10875_2022_1312_MOESM1_ESM.png]

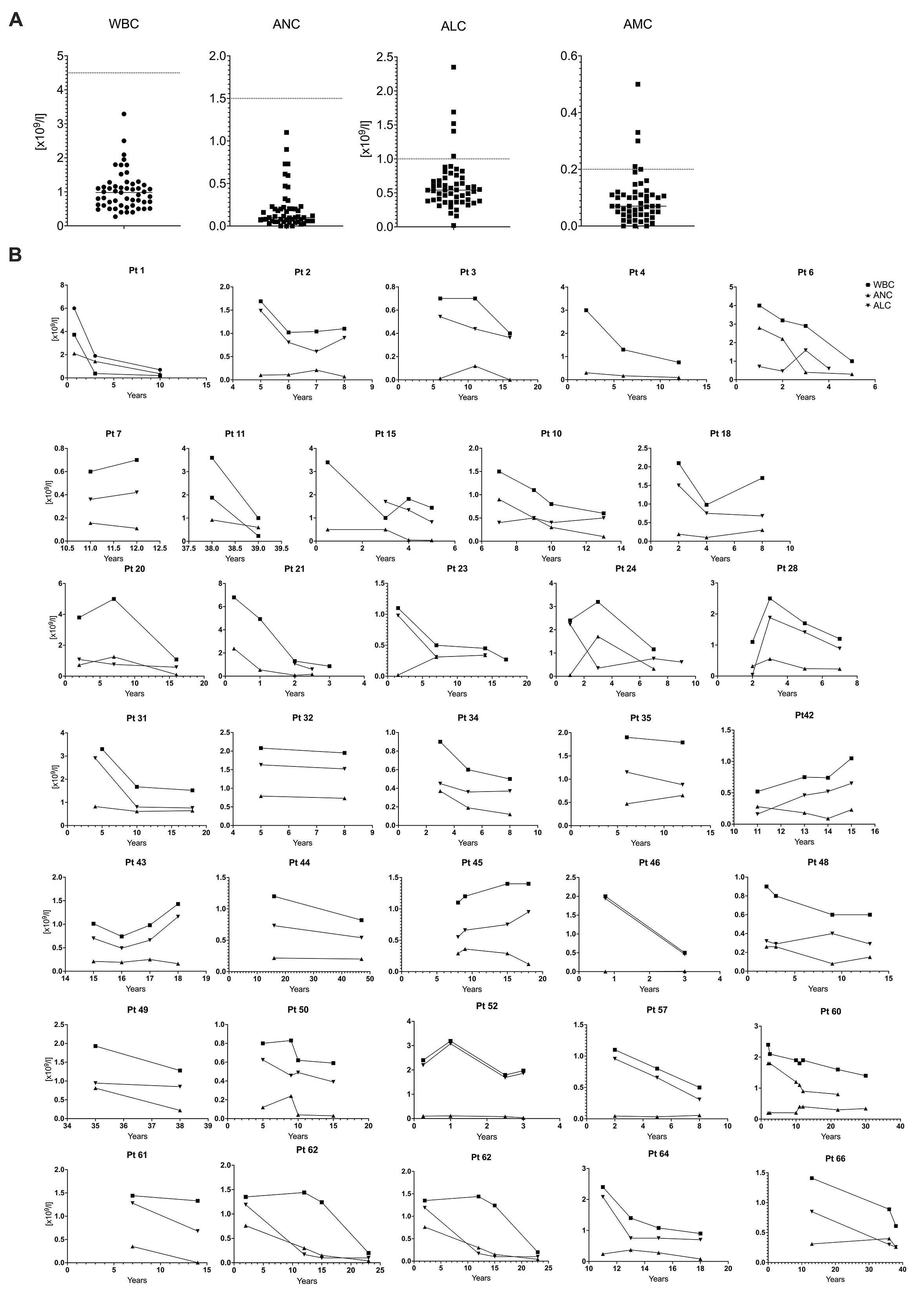

Supplement: Supplementary file 3 — High Resolution Image (PNG 556 kb) [file 10875_2022_1312_Fig6_ESM.png]
