# Supplementary material for: Disease Progression of WHIM Syndrome in an International Cohort of 66 Pediatric and Adult Patients
Source: J Clin Immunol. 2022 Aug 10;42(8):1748–65. doi: 10.1007/s10875-022-01312-7 (PMC9700649; doi:10.1007/s10875-022-01312-7)
Supplement: Supplementary file 6 — (DOCX 14 kb) [file 10875_2022_1312_MOESM5_ESM.docx]

| Oligo | | DNA |
| --- | --- | --- |
| Name | | Sequence (5'-3') |
| T318N fs*26 | F | CCA GCA CGC ACT CAA CCT CTG TGA GCA GAG |
| T318N fs*26 | R | CTC TGC TCA CAG AGG TTG AGT GCG TGC TGG |
| S319L fs*2 | F | CAG CAC GCA CTC ACT CTG TGA GCA GAG G |
| S319L fs*2 | R | CCT CTG CTC ACA GAG TGA GTG CGT GCT G |
| S324F fs*21 | F | GTG AGC AGA GGG TTC CTC CAG CCT CAA GAT C |
| S324F fs*21 | R | GAT CTT GAG GCT GGA GGA ACC CTC TGC TCA C |
| S324P fs*42 | F | CTC TGT GAG CAG AGG TCC AGC CTC AAG ATC |
| S324P fs*42 | R | GAT CTT GAG GCT GGA CCT CTG CTC ACA GAG |
| R322Q fs*22 | F | CAC CTC TGT GAG CCA GAG GGT CCA GCC |
| R322Q fs*22 | R | GGC TGG ACC CTC TGG CTC ACA GAG GTG |
| L326Q fs*17 | F | GCA GAG GGT CCA GCC AAG ATC CTC TCC AAA G |
| L326Q fs*17 | R | CTT TGG AGA GGA TCT TGG CTG GAC CCT CTG C |
| K327R fs*17 | F | GAG GGT CCA GCC TCA GAG ATC CTC TCC AAA G |
| K327R fs*17 | R | CTT TGG AGA GGA TCT CTG AGG CTG GAC CCT C |
| V340L fs*27 | F | GGT GGA CAT TCA TCT CTG TTT CCA CTG AGT C |
| V340L fs*27 | R | GAC TCA GTG GAA ACA GAG ATG AAT GTC CAC C |
| S346* | F | CTG AGT CTG AGT AAG TTT TCA CTC C |
| S346* | R | GGA GTG AAA ACT TAC TCA GAC TCA G |
| S339L*27 | F | GAGGTGGACATTCTCTGTTTCCACTG |
| S339L*27 | R | CAGTGGAAACAGAGAATGTCCACCTC |
